# From air embolism to acute respiratory distress syndrome: A rare complication in a posterior fossa tumour excision — A case report

**DOI:** 10.1016/j.ijscr.2025.111836

**Published:** 2025-08-18

**Authors:** Jawdat M. Alali, Umm E. Amara, Umm E. Nashrah, Firdous E. Ummunnisa, Mahmoud Tabouni, Nissar M. Shaikh

**Affiliations:** a Surgical Intensive Care Unit, Hamad Medical Corporation (HMC), Doha, Qatar; b Deccan College of Medical Sciences, Hyderabad, India; c Halima Al-Tamimi OBGY Clinic, Doha, Qatar; d Internal Medicine Dept, Hamad Medical Corporation (HMC), Doha, Qatar

**Keywords:** ARDS, Craniotomy, Pleural effusion, SIRS, Vascular air embolism, Intensive care management

## Abstract

**Introduction and importance:**

Vascular air embolism (VAE) is a rare but potentially life-threatening complication, especially during neurosurgical procedures in the sitting position. While intraoperative VAE is well-documented, its progression to systemic inflammatory response syndrome (SIRS), massive pleural effusion, and acute respiratory distress syndrome (ARDS) is exceedingly rare.

**Case presentation:**

A 32-year-old woman undergoing cerebellopontine angle schwannoma excision developed intraoperative VAE. Despite successful initial management, she progressed to SIRS, bilateral pleural effusions, and ARDS. She required invasive ventilation, bilateral pleural drainage, vasopressor support, and advanced hemodynamic monitoring with PiCCO. Gradual improvement was observed with supportive care, and she was successfully extubated and discharged.

**Clinical discussion:**

This case illustrates how micro air emboli can trigger severe inflammatory and pulmonary responses, mimicking sepsis and cardiac failure. Capillary leak and pulmonary endothelial injury are key mechanisms.

**Conclusion:**

VAE can lead to serious complications including ARDS and SIRS. Early recognition and aggressive supportive management are essential for recovery.

## Highlights

• Vascular air embolism occurred during CP angle tumour excision in sitting position.
• The embolism triggered systemic inflammatory response syndrome with high-grade fever.
• The patient developed ARDS with massive bilateral pleural effusion requiring drainage.
• PiCCO monitoring guided fluid management and vasopressor/inotrope weaning.
• Early recognition and multidisciplinary critical care led to full patient recovery.

## 1 Introduction

Vascular air embolism (VAE) is a life and organ-threatening clinical entity [1]. VAE can be due to either a direct communication between the source of air and vasculature or it can occur due to a pressure gradient favouring suction of air into the circulation [2]. VAE may occur due to surgical intervention, trauma or any vascular intervention. Neurologic and otolaryngology procedures may cause more VAE compared to other surgical procedures. The risk of occurrence of VAE in neurosurgical interventions is due to the sitting position required for intervention [3].

We report a case of VAE, causing systemic inflammatory response syndrome (SIRS), massive bilateral pleural effusion, and acute respiratory distress syndrome (ARDS).

This case report has been reported in line with the SCARE checklist [4].

## 2 Case presentation

A 32-year-old female with a right-sided cerebropontine (CP) angle acoustic Schwannoma was admitted for tumour excision. She was obese (BMI 38) but had no other significant medical, surgical, family or allergic history. On the day of surgery, she was induced with intravenous anaesthesia and maintained with a mixture of sevoflurane, oxygen and air along with intravenous infusions of propofol and remifentanil and timed boluses of rocuronium. Electrocardiogram (ECG), invasive blood pressure, pulse oximetry (SpO_2_), end tidal carbon dioxide (EtCO_2_), urine output and ventilatory parameters were monitored intraoperatively and the patient was placed in a sitting position. After opening the dura, the EtCO_2_ suddenly dropped from 40 to 15, the patient desaturated to 86 % and became hypotensive. VAE was suspected. Immediately, 2 to 3 mL of air were aspirated from the CVC (central venous catheter). Further bilateral jugular venous compression, spraying the surgical field with normal saline, and increasing the fraction of inspired of oxygen concentration (FIO2) to 1 were performed. The patient responded to these treatment measures, and the surgery was then completed. At the end of the surgery the patient developed two more episodes characterised by sudden drops in Etco_2_ with desaturation.

**Fig. 1:**
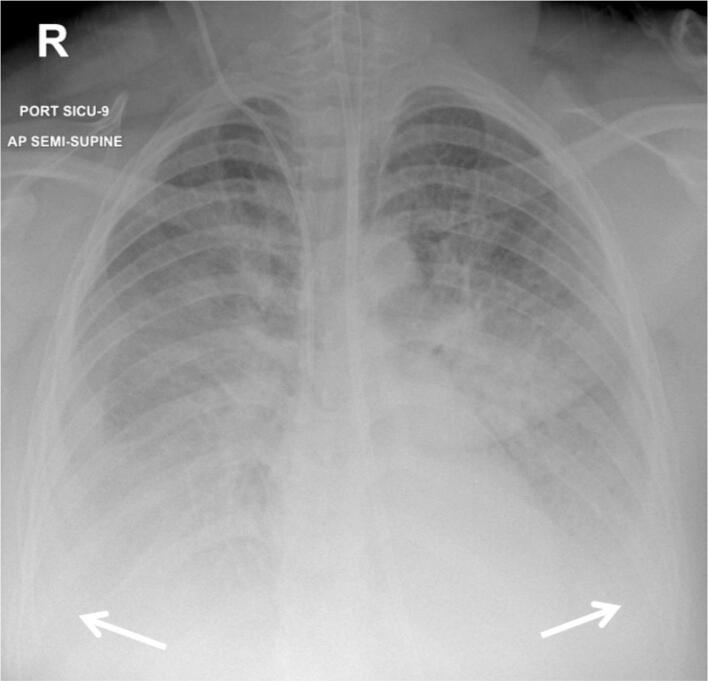
Chest x-ray showing ARDS and pleural effusion: Bilateral scattered airspace opacities. Obliterated both costophrenic angles (white arrows).

Post operatively, the patient was transferred to the SICU ventilated with high FiO2 and a PEEP of 10 cmH_2_O and started on 0.08 μg/kg/min of noradrenaline. A chest x-ray (Fig. 1) and ultrasound revealed pulmonary oedema suggested by the presence of bilateral infiltrates and many B-lines, respectively. An arterial blood gas (ABG) revealed the following: pH 7.27, Pco_2_ 53, Po_2_ 45, lactic acid 3.5 mmol/L P/F (Pa0_2_/Fio_2_) 87. After a few hours, further changes to the ventilator settings were required, namely the respiratory rate was increased to 20 breaths/min and positive end expiratory pressure (PEEP) to 14 cmH_2_O but the FiO_2_ remained unchanged at 1.

Point of care ultrasound (POCUS) demonstrated good cardiac contractility. She was sedated and paralysed with propofol, remifentanil, and rocuronium. Her pupils were equal and reactive to light bilaterally. Her laboratory work-up revealed leucocytosis and thrombocytosis, she was highly febrile (39.6 °C) with supraventricular tachycardia. Septic markers and workup were taken, and she was started on tazobactam/piperacillin antibiotic empirically. She required noradrenaline to maintain hemodynamic stability.

**Fig. 2:**
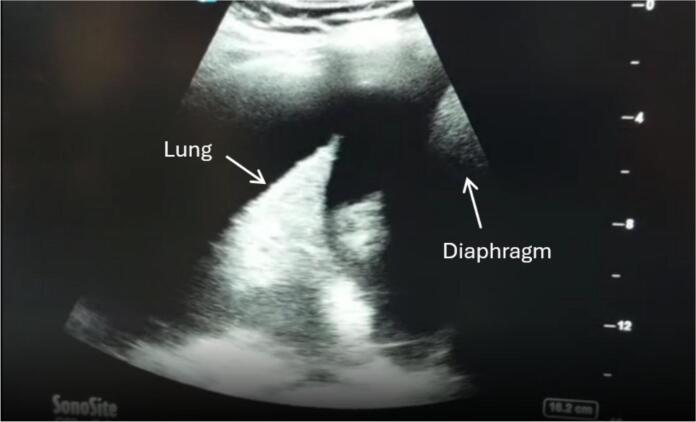
POCUS showing massive pleural effusion: Anechoic area located above the diaphragm and around the lung, indicating the presence of free fluid within the pleural space.

A repeat echocardiogram suggested no abnormalities. A repeat chest x-ray exhibited bilateral diffuse infiltrates and signs of bilateral pleural effusion which was confirmed on a chest ultrasound as seen in (Fig. 2).

**Fig. 3:**
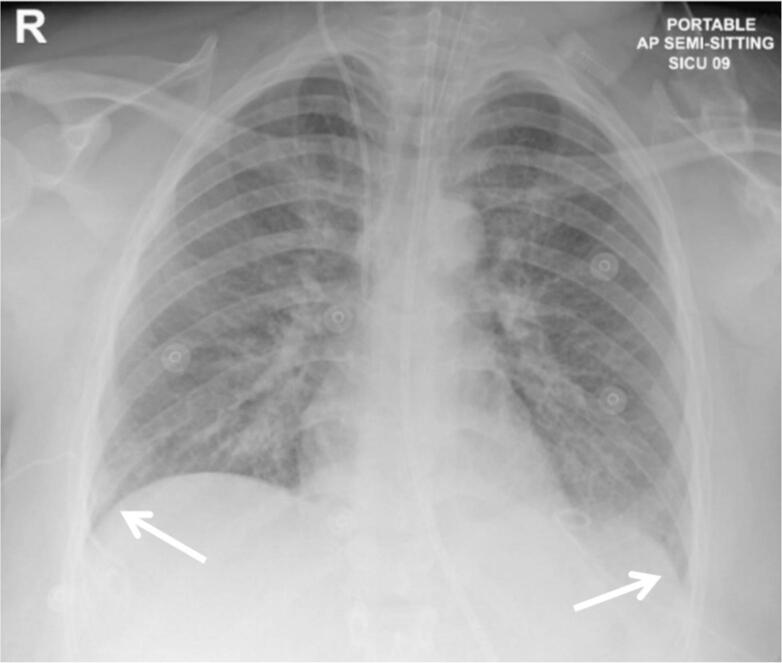
Chest x-ray after drainage of pleural effusion: No significant interval changes about the lung field. Both costophrenic angles are clearer in comparison with previous x-ray (white arrows). Bilateral chest drains in place.

This was treated using pigtail catheters inserted on both sides of the chest which drained around 4 L of transudative fluid within 24 h. Chest x-ray improved (Fig. 3). Since she remained febrile up to 40 °C, pharmacological and surface cooling were commenced.

Pulse integrated continuous cardiac output (PiCCO) catheter was inserted and it revealed high extravascular lung water index (EVLWI), systemic vascular index (SVRI) and pulmonary vascular permeability index (PVPI) as seen in Table 1.

**Table 1:** PiCCO findings and medications given at the time:

Day	CI	SVRI	EVLWI	PVPI	GEDVI	On
1	1.5	3800	16	4.1	540	Noradrenalin + furosemide
Evening	2.1	2900	11.9	3.2	550	Noradrenalin + milrinone + furosemide
2	2.6	2000	10.2	2.8	450	Milrinone
3	2.4	1437	8.9	1.8	722	Milrinone + frusemide

CI: cardiac index (L/min/m^2^).

SVRI: systemic vascular resistance index.

EVLWI: extravascular lung water index.

PVPI: pulmonary vascular permeability index.

GEDVI: global end-diastolic volume index.

Therefore, milrinone was started and she was weaned off noradrenaline. Echocardiogram and cardiac biomarkers were within normal ranges and there were no changes in her ECG. The patient remained febrile at 40 °C and tachycardiac at 140 beats/min. Day 2, the patient showed improvement clinically; however, she was still febrile. Her FiO_2_ requirements dropped to 0.4. Her PEEP was reduced to 12 cmH2O and her ABGs also showed improvement with a PO2 of 93 and P/F of 300, although her lactate levels remained high (3.1 mmol/L). By day 3, furosemide, milrinone and neuromuscular blockers were stopped as the patient remained hemodynamically stable. On day 4 patient had a few episodes of desaturation and remained intubated while sedation was changed to dexmedetomidine. She was afebrile, and septic markers and septic workup (including blood cultures) were negative. She required one session of proning due to high PO_2_ requirement. Serial chest x-rays showed gradual resolution of the bilateral diffuse infiltrates. She was weaned from the ventilator and extubated as she was awake and obeying commands by day 6. She initially remained on NIV (non- invasive ventilation) and then shifted to nasal cannula and room air. On day 8, all invasive lines were removed, and she was started on oral diet. Antibiotics were de-escalated by Day 10 when she was transferred to surgical ward. From there she was discharged home and followed up at 4 weeks and 3 months in an outpatient clinic. She was doing fine without any issues. Although the patient fulfilled the diagnostic criteria for SIRS, including hyperthermia, tachycardia, leukocytosis, and hypotension requiring vasopressors, there was no microbiological evidence of infection, and blood cultures were negative. Her rapid clinical improvement with supportive management and absence of a definitive infectious source point toward a non-infectious SIRS, likely triggered by venous air embolism and secondary ARDS. Therefore, a diagnosis of sepsis could not be substantiated, and her inflammatory response was best classified as severe SIRS due to a traumatic vascular insult rather than infection.

## 3 Discussion

Vascular air embolism (VAE) in the sitting and prone position during neurosurgical intervention occurs as a result of pressure gradient as the surgical field is above the level of the heart which facilitates air entry into the circulation, and the intraoperative embolism is detected mainly by sudden drop in end tidal carbon dioxide drop and increased requirements of inspired oxygen whereas in an awake patient with severe VAE, respiratory symptoms, particularly dyspnea, is universal [5].

Our patient had a sudden drop in EtCO_2_ and immediately air was aspirated from the CVC. Wong et al. reported similar findings *via* transesophageal echocardiography [6]. Imaging studies including computed tomography (CT), echocardiogram and x-rays are typically normal as it is known that the air is rapidly absorbed from the circulation, but in cases of continuous embolization of air end organ damage can be caused and air can be visualized and further complications like ARDS and SIRS can occur [6]. Air micro-emboli lodged in the pulmonary circulation interact with endothelial cells and initiate thrombo-inflammatory responses through platelet activation, complement cascade stimulation, and neutrophil recruitment, leading to endothelial injury, increased vascular permeability, and systemic inflammatory activation [7].

In posterior fossa surgery, particularly in the prone or semi-sitting position, careful intraoperative anesthetic monitoring is essential for early detection and management of air embolism. As described by Akano and Schlichter, tools such as end-tidal CO₂ (EtCO₂) monitoring and prompt aspiration through central venous catheters are critical—both of which were utilized effectively in our case [8]. Günther et al. analyzed the incidence and severity of venous air embolism (VAE) in cranial surgeries performed in the sitting position, showing that VAE is relatively common but often subclinical when using sensitive monitoring methods [9]. In contrast, our patient, also in the sitting position, developed clinically significant VAE, evidenced by a sudden EtCO₂ drop and air aspiration from the central line, which progressed to ARDS and SIRS—highlighting a more severe and rare outcome.

Mahajan et al. reported VAE during endoscopic transsphenoidal pituitary surgery, with intraoperative EtCO₂ drop as a key sign [10]. Similarly, our patient developed VAE during posterior fossa surgery in the sitting position, which increases embolism risk. Unlike their case, our patient progressed to ARDS and systemic inflammation. Both highlight the importance of careful monitoring and prompt management in sitting-position neurosurgeries.

Secondary injury from micro air embolism causes organ dysfunction; the air bubbles in pulmonary microcirculation are associated with local endothelial injury with aggregation of neutrophils, platelets, fibrin and lipid droplets at the gas fluid interface, further activation of complement system causes more endothelial damage. These all lead to the non-cardiogenic pulmonary oedema or ARDS, bronchoconstriction, alveolar consolidation, increased physiological dead space and ventilation perfusion mismatch [11,12]. In our patient there was capillary leakage (PVPI) and non-cardiogenic pulmonary oedema (ARDS). A similar mechanism caused systemic inflammatory response syndrome; most of the evidence comes from the experimental and decompression reporting, after bubble contact and endothelial injury there is release of endothelial micro platelets, activation of platelets, leucocytes, erythrocytes and neutrophils lead to diffuse systemic inflammatory response [13]. Our patient also had leukocytosis, thrombocytosis, and was febrile, which is likely explained by SIRS as septic workup was negative. Kapoor et al. also reported a case of air embolism causing severe systemic inflammatory response syndrome massive pleural effusion requiring drainage [14]. The similar situation occurred in our patient may have also occurred due to severe SIRS probably due to micro air emboli and bubbles.

Fitchet et al. reported a case of air embolism causing pulmonary oedema that mimicked left ventricular failure and resulted in bilateral pleural effusions [15]. Similarly, Clark et al., in their case report, described air embolism leading to acute respiratory distress syndrome (ARDS) and pleural effusion, in contrast to this case, where the pleural effusion was exudative due to capillary leakage and increased microvascular permeability associated with ARDS, our patient developed a transudative pleural effusion, likely resulting from increased hydrostatic pressure and fluid overload secondary to venous air embolism-induced cardiopulmonary compromise, rather than direct endothelial injury [12].

## 4 Future implications

This case highlights the need for heightened intraoperative vigilance during neurosurgical procedures in the sitting position due to the risk of vascular air embolism (VAE). It suggests a potential role for integrating early use of advanced hemodynamic monitoring tools, such as PiCCO, in patients with suspected microvascular air embolism to guide fluid management and detect capillary leak syndrome. Guidelines may also benefit from emphasizing a broader differential diagnosis for postoperative fever and respiratory failure, including VAE-induced systemic inflammatory response syndrome (SIRS) and ARDS.

### 4.1 Take-away lessons

• Micro air emboli can cause severe complications including SIRS, ARDS, and pleural effusion even after apparent initial stabilization.
• PiCCO-guided therapy was crucial in managing fluid status and improving outcomes.
• The case underscores the importance of prompt multidisciplinary coordination involving neurosurgery, critical care, and respiratory therapy.

### 4.2 Strengths

• This report describes a rare progression of VAE to ARDS and SIRS in an immunocompetent patient with no cardiac dysfunction, filling a gap in existing literature.
• The use of PiCCO provided objective, real-time insights into pulmonary permeability and fluid overload, guiding individualized management.
• The case had high multidisciplinary relevance, involving neurosurgery, anesthesiology, intensive care, and radiology, showcasing effective collaboration in a complex postoperative course.

### 4.3 Weaknesses and limitations

• The single-patient nature of the case limits generalizability; the rare complication profile may not reflect broader population risks.
• Continuous echocardiographic monitoring during the intraoperative period might have provided earlier detection and more direct visualization of VAE.

## 5 Conclusion

Micro bubbles due to air embolism can cause acute respiratory distress syndrome with massive bilateral pleural effusion, leucocytosis, thrombocytosis and high-grade fever due to systemic inflammatory response syndrome and capillary leak.

## CRediT authorship contribution statement

**Jawdat M. Alali, MD:** Contributed to the study concept and literature review, supervised patient management in the SICU, and reviewed and revised the manuscript critically for intellectual content.
**Umm E Amara, Medical Student:** Assisted in literature review, and drafting of the initial manuscript.
**Umm E Nashrah, Medical Student:** Assisted in literature review, and contributed to manuscript writing and formatting.
**Firdous E Ummunnisa, MD:** Contributed to clinical interpretation of the patient's course and provided revisions to the manuscript content.
**Mahmoud Tabouni, MD:** Corresponding author; involved in drafting of the initial manuscript, overall coordination and final approval of the submitted version.
**Nissar M Shaikh, MD:** Participated in patient care during SICU stay, contributed to data interpretation and manuscript writing and review.

All authors have read and approved the final manuscript and agree to be accountable for all aspects of the work.

## Consent

Written informed consent was obtained from the patient for publication and any accompanying images. A copy of the written consent is available for review by the Editor-in-Chief of this journal on request.

## Ethical approval

This case report was conducted in accordance with institutional policies. Ethical approval was not required as per the guidelines for single patient case reports. Written informed consent for publication was obtained from the patient.

## Guarantor

Dr. Mahmoud Tabouni accepts full responsibility for the integrity of the work, had full access to the data, and controlled the decision to publish this case report.

## Research registration number

This study does not qualify as a First in Man study and thus does not require registration under that category.

## Declaration of Generative AI and AI-assisted technologies in the writing process

During the preparation of this work, we used ChatGPT (OpenAI) and Grammarly in order to assist with language refinement, grammar correction, and improving clarity and coherence of the text. After using these tools, we reviewed and edited the content as needed and take full responsibility for the content of the publication.

## Funding

This research did not receive any specific grant from funding agencies in the public, commercial, or non-profit sectors.

## Declaration of competing interest

The authors declare that they have no conflicts of interest related to this work.
